# An interpretable method for automated classification of spoken transcripts and written text

**DOI:** 10.1007/s12065-023-00851-1

**Published:** 2023-05-04

**Authors:** Mattias Wahde, Marco L. Della Vedova, Marco Virgolin, Minerva Suvanto

**Affiliations:** 1 Chalmers University of Technology, 412 96 Gothenburg, Sweden; 2 Evolutionary Intelligence Group, Centrum Wiskunde and Informatica, Science Park 123, Amsterdam, 1098 XG The Netherlands

**Keywords:** Text classification, Natural language processing, Interpretable methods

## Abstract

We investigate the differences between spoken language (in the form of radio show transcripts) and written language (Wikipedia articles) in the context of text classification. We present a novel, interpretable method for text classification, involving a linear classifier using a large set of $n-$gram features, and apply it to a newly generated data set with sentences originating either from spoken transcripts or written text. Our classifier reaches an accuracy less than 0.02 below that of a commonly used classifier (DistilBERT) based on deep neural networks (DNNs). Moreover, our classifier has an integrated measure of confidence, for assessing the reliability of a given classification. An online tool is provided for demonstrating our classifier, particularly its interpretable nature, which is a crucial feature in classification tasks involving high-stakes decision-making. We also study the capability of DistilBERT to carry out fill-in-the-blank tasks in either spoken or written text, and find it to perform similarly in both cases. Our main conclusion is that, with careful improvements, the performance gap between classical methods and DNN-based methods may be reduced significantly, such that the choice of classification method comes down to the need (if any) for interpretability.

## Introduction

Currently, the field of natural language processing (NLP) is dominated by large-scale statistical language models (LLMs) consisting of deep neural networks (DNNs). LLMs that use the transformer DNN architecture [1] offer state-of-the-art performance, as evidenced by systems such as BERT [2] and DistilBERT [3], GPT-2, GPT-3 [4], and the much-publicized ChatGPT. On the other hand, DNN-based systems also come with drawbacks [5, 6], one of the most important being their opaque nature: In most cases, it is near-impossible to determine precisely *how* such systems make decisions.

Now, in many cases, that does not matter. For example, when chatting on everyday topics or classifying, say, movie or restaurant reviews, the stakes are low and occasional errors do not matter much. However, there are also situations that involve high-stakes decision-making [6], for example when a person is conversing with an artificial system about a serious topic (e.g., a medical diagnosis, legal advice, or financial matters). Another example is the problem of classifying a particular text to determine whether it has offensive content, contains biases against minorities, or represents fake news. In *those* situations, errors may have very serious consequences. Here, it should be noted that the problem is not the error in itself; after all, any decision-maker, whether human or artificial, can sometimes make errors. Instead, the problem that specifically pertains to the DNN-based LLMs is their limited interpretability. While the state-of-the-art LLMs generally exhibit very good average performance, they sometimes make catastrophic, inexplicable, and inscrutable errors. A recent example is ChatGPT that, despite its impressive performance, also can fail spectacularly while at the same time even offering bogus supporting arguments for preceding incorrect statements, typically with near-perfect grammar, often making it hard for an uninitiated human observer to detect the error [7]. Although many approaches exist to explain opaque models (including DNN-based LLMs), these remain limited in that they only represent partial explanations (e.g., attention maps) or approximate explanations (e.g., LIME and SHAP [8, 9]).

Turning now to the specific case of text classification, which is the topic of this paper, BERT (and models derived from it, such as DistilBERT) is a cornerstone model from which many other LLMs have followed in recent years [10]. In particular, as a result of its training, where one modality involved next-sentence prediction, i.e., determining whether or not a given sentence B logically follows another sentence A, the output corresponding to (the embedding of) the [CLS] (classification) token, which is preprended to every input token sequence, can be seen as a condensed representation of a string of tokens (e.g., a sentence). This part of BERT’s output can therefore be used for classification purposes, typically by adding a feedforward layer with softmax applied to its output, and then fine-tuning the system over a training data set with known labels, whereby the weights of the feedforward layer are optimized, while the weights of BERT itself are (optionally) fine-tuned.

This setup, involving transfer learning applied to BERT or one of its derived versions, has been applied in many classification problems; see, e.g., [11–14]. In such problems, it is common to compare BERT’s performance with a set of classical benchmark models, such as linear regression (with thresholding to turn it into a classifier), logistic regression, naïve Bayesian classifiers, decision trees, support vector machines, and so on. The typical finding is that the DNN-based models outperform the classical ones by a large margin, not seldom 5–10 percentage points or even more. This state of affairs presents a dilemma to an end-user, assuming that the classification task at hand pertains to a high-stakes decision-making problem: Should one use a classical model that typically offers a high degree of interpretability (albeit somewhat different between the different models) but inferior performance, or should one use a DNN-based system that offers better performance but very limited interpretability?

In recent years, considerable efforts have been made to define, tweak, and optimize DNN-based text classifiers [13]. By contrast, the classical benchmark methods are generally given very little attention: To the extent that they are mentioned at all, they are often used off-the-shelf, just providing a backdrop against which the DNNs are compared. The large performance difference, in favor of the DNN-based text classifiers, is therefore perhaps not very surprising, but it also leads us to an interesting question, which we will attempt to answer in this paper: Is it possible to improve the performance of the classical methods, either by adjusting the features used or the methods themselves, so as to reduce or even close the performance gap, while maintaining a high degree of interpretability?

Here, we will consider a specific (binary) classification task involving a newly generated data set with texts (sentences) belonging to either of two classes: Transcripts of spoken utterances (Class 0), and texts that were in written form from the beginning (Class 1). More specifically, the sentences in Class 0 are taken from publicly available transcripts from radio shows, whereas the sentences in Class 1 are taken from Wikipedia articles. Expressed differently, one may say that Class 0 is dominated by sentences in informal, spoken language, whereas Class 1 primarily contains sentences with more formal language.

In order to investigate the question posed above, almost any text classification task would do. However, we will also investigate a second question, for which the data set just mentioned is well suited. During its development, BERT was (pre-)trained using two large data sets: Wikipedia (2.5 billion tokens) and BooksCorpus (800 million tokens). Now, using the class definitions above, text in Wikipedia consists primarily of sentences that would belong to Class 1 (written). There *are* sentences belonging to Class 0 in Wikipedia articles as well, for example, quotes and excerpts of dialogue, but it is reasonable to assume that they represent only a small fraction of Wikipedia’s content. The BooksCorpus is not described in detail by the original authors, but it nevertheless contains a large amount of informal spoken language. However, as the BooksCorpus is considerably smaller than Wikipedia, one may be concerned that BERT’s ability to deal with spoken, informal language (e.g., in fill-in-the-blank tasks) may be less good than its ability to handle formal, written language. This question is made even more pertinent given the somewhat murky details of the (original) BooksCorpus, as discussed in [15].

The main result of this work is that, after feature selection and optimization, a custom linear classifier is able to obtain a classification performance approaching that of DNN-based models (in this case DistilBERT), while maintaining interpretability, i.e., providing an easily human-understandable description of the different features and their contribution to the overall classification. We also provide a tool (see Sect. 5.4) making it possible even for a non-specialist human observer to understand how the classifier made its decision. An additional output is the data set itself, which we freely provide for use by researchers^1^ interested in studying the differences between spoken transcripts and written, formal text. Finally, we show that, perhaps somewhat contrary to our expectations, DistilBERT is, in fact, more or less equally good at filling in blanks in either written text (Class 1) or transcripts of spoken utterances (Class 0).

The paper is organized as follows: In Sect. 2, we describe some related work. Section 3 describes our data set, and our custom linear classifier is presented in Sect. 4. In Sect. 5 we give our results, and then a discussion follows in Sect. 6. The conclusions are given in Sect. 7.

## Related work

To our knowledge, this study is the first that considers the issue of comparing spoken utterances and written text, in the context of classification over a large data set. As mentioned above, we will show that our classifier exhibits good performance, approaching that of the DNN-based DistilBERT classifier, while maintaining interpretability. This is in stark contrast to the often very large reported performance difference between, on the one hand, DNN-based classifiers and, on the other hand, any other method. For example, in the case of text sentiment analysis using the IMDB data set of movie reviews, the best-performing DNN-based classifiers [16, 17] have a reported accuracy in the range of around 0.95 to 0.97, whereas classical methods such as kNN classifiers, naïve Bayesian classifiers, support vector machines (SVMs), logistic regression, and so on, typically do not exceed an accuracy of 0.9, thus indicating a difference in accuracy of around 0.05$-$0.07. Similarly, in the context of fake news detection, DNN-based methods outperform methods based on decision trees and SVMs by a difference in accuracy of up to about 0.30 depending on the data set [18]. Another example, involving fake news detection related to Covid-19, is given in [19], where a version of BERT achieved an accuracy very close to 1, compared to an accuracy of around 0.94 for a method combining a naïve Bayesian classifier and an SVM [20].

It should be noted that, possibly because of the results described above, recent papers on text classification barely even *mention* any approaches other than black box, DNN-based methods, comparing them to each other, see e.g. [13] and the references therein. While the DNN-based methods undoubtedly are leading in performance at present, there may be other dimensions to consider as well, for example the level of interpretability of a classifier, an aspect where the complex and highly non-linear DNN-based classifiers are severely limited. The classical methods are generally less opaque, but were not explicitly designed for interpretability that, arguably, was not an important issue before the advent of large DNN-based text classifiers.

In the context of the interpretation of text classification methods, [21] propose an approach similar to ours regarding the visualization of the contribution of individual words. However, their approach is based on a secondary method (i.e., layer-wise relevance propagation, LRP) that aims to explain the output of another classification method (e.g., a CNN) rather than providing an explicitly interpretable recipe (as our method does) for how the emphasized words are combined in order to make the classification.

## Data

For text classification of the kind considered in this paper, it is assumed that the data sets consist of *k* sentences, of which $k_0$ belong to Class 0 and $k_1$ to Class 1, i.e., the two classes defined in Sect. 1. In the case of the specific data set used here, sentences belonging to the *spoken* class (Class 0) were generated from publicly available data sets with transcripts from radio shows, namely several shows from National Public Radio (NPR) and the radio show *This American Life*. Texts from those sources were split into individual sentences, which were then added to the data set. For Class 1, sentences were generated from a large number of randomly selected Wikipedia pages (excluding special, redirect, disambiguation, and list-of pages), again splitting the text into individual sentences. Now, after visual inspection of the data set, it was deemed that very short sentences should be excluded, as there is often no possibility of reliably assigning a class label for such sentences; many short sentences could very well be seen as belonging to either class. In the end, the lower limit was set at 5 tokens (including the end-of-sentence marker, which is tokenized as well; see Sect. 4.1.1 below).

In total, after removing short sentences, 13,640,458 sentences were retained, of which 6,374,487 in Class 0 and 7,265,971 in Class 1. Next, the data set was split into three subsets, a training set (with approximately 5/7 of the total number of sentences), a validation set (1/7), and a test set (1/7). Thus, the training set contained 9,743,188 sentences (of which 4,553,205 in Class 0 and 5,189,983 in Class 1), the validation set 1,948,639 sentences (of which 910,641 in Class 0 and 1,037,998 in Class 1), and the test set 1,948,631 sentences (of which 910,641 in Class0 and 1,037,990 in Class1); see also Table 1.

**Table 1:** Data set split information

Subset	Class 0	Class 1	Total
Training	0.3336	0.3802	0.7138
Validation	0.0668	0.0761	0.1429
Test	0.0668	0.0761	0.1429

The fraction of samples in each of the three subsets, and for each class label

### Spoken vs. written text

In order to measure differences in token frequencies between spoken and written language, the *spokenness* measure (*s*) is here introduced as

1 $$\begin{aligned} s(t) = \log _{10}\frac{f_{s}(t)}{f_{w}(t)}, \end{aligned}$$

where *t* denotes a token, $f_{s}(t)$ denotes its relative frequency among the spoken sentences (Class 0) and $f_w(t)$ its frequency among the written sentences (Class 1). The measure is computed only for those tokens that appear at least once in both sets. The left column of Table 2 shows some examples of tokens with strongly positive spokenness, and the right column shows some examples with strongly negative spokenness values, when computed over the training set defined above. As can be seen from the table, the relative token frequency differs by a factor 100 or more for some tokens.

**Table 2:** Examples of spokenness values for different tokens

Token	Spokenness	Token	Spokenness
how’d	2.3385	relegation	$-$3.0993
who’ve	2.2970	footballer	$-$2.9392
yeah	2.2871	duchy	$-$2.8519
here’s	2.1295	ventral	$-$2.7050
they’re	2.0272	uncredited	$-$2.6415

Examples of tokens with strongly positive spokenness (left column) and strongly negative spokenness (right column), in the training set used here; see also Eq. 1. Note that misspelled words and proper nouns, e.g., names, are not shown in the list (but are included in the tokens generated by our tokenizer)

The fact that many words have a spokenness value very different from zero (either negative or positive) may cause some concern regarding the performance (in any NLP task) of language models trained over predominantly written data sources. An example is BERT, for which a large majority of the training data came from written sources (see Sect. 1). On the other hand, it is likely that the number of words considered in spoken dialogue is much smaller than the number of tokens used in written dialogue, perhaps making it easier to train a language model for spoken text. Indeed, for our data set, using the tokenization method described in Sect. 4.1.1 below, the spoken data (in the training set) contributes 128,689 tokens with at least 3 instances or more, whereas the written set contributes 439,641 tokens. There is of course a considerable overlap between these two token sets: The total number of tokens with at least 3 *total* instances (regardless of the class) is equal to 481,452.

## Method

This section describes the proposed method in general terms, i.e., independent of the data set used, starting with feature generation. Next, the structure of the classifier is described and then the optimization method.

### Classification features

In the proposed method, bag-of-words-style (or, rather, bag-of-n-gram) features are used. Thus, the features are defined as the counts (number of instances) of $n-$grams in the text or utterance that is to be classified.

#### Tokenization

Starting with the unigram features ($n=1$), we have written a custom, very inclusive tokenizer, i.e., one that generates a very large set of tokens (i.e., unigrams), by keeping words as they are, essentially just splitting the data sets on the space character, and treating (some) special characters, e.g. parentheses, quotation marks, etc. as separate tokens. The number of tokens thus generated is generally orders of magnitude larger than, say, the 30,522 tokens used in standard BERT, and a bit larger than the number of tokens generated by the scikit-learn standard tokenizer.

#### Token sequences

The method also makes use of $n-$grams with $n > 1$. In principle, the feature generation could involve several values of *n*, up to a maximum value which is here denoted $n_\mathrm{{max}}$. However, in practice, it is sufficient to stop the feature generation at a small value of *n*, in our case $n=2$ (bigrams), as illustrated in Sect. 5 below. Once a text has been tokenized, from the *m* tokens, the $m-n+1$ possible $n-$grams are generated, and the number of instances of each $n-$gram is counted, i.e., the same procedure as for the unigrams.

#### Generating the feature set

When generating a feature set, the tokenization is carried out first, resulting in a list of tokens for each text. In preparation for optimization, the training set is tokenized, and the resulting tokens (unigrams) are stored in a feature set, along with information about the number of instances in each of the two classes. Next, all bigrams are generated by considering consecutive tokens. In the specific case considered here, the training set consists of *k* individual sentences, so that the total number of bigrams is equal to $m - k$, where *m* is the total number of tokens in the training set. As in the case of the unigrams, the number of instances (in each class) is noted for each bigram. The procedure can then, in principle, be extended to include trigrams, and so on.

From this large, preliminary feature set, rare features are removed to avoid overfitting during optimization, by simply counting the number of occurrences of each feature in the training set, and then removing those features that appear less than *p* times in that set, where *p* is an integer parameter. After that, the unigrams, bigrams, and so on, are sorted in alphabetical order to form the feature set.

Next, both the training set and the validation set are indexed, which can be done with an efficient binary search, resulting in a feature index list, such that features can be accessed by simple indexing, that is, an O(1) process, with minimal overhead. Note that, when indexing the validation set, in some cases there may be unigrams, bigrams, and so on that do not appear in the training set, and are therefore not included in the feature set. In such cases, the index is set to -1, and the corresponding feature will be ignored by the classifier (see below). However, due to the inclusive nature of the feature set, the vast majority of features in the validation set can also be found in the training set.

### Classifier

The classifier has a simple, essentially linear, structure, but with a length-dependent adjustment term. Let $f_i$ denote the number of instances of feature *i* in a given text, where the features are the $n-$grams described above, and let *V* denote the total number of distinct features in the feature set. Furthermore, let $L_1$ denote the number of tokens (unigrams) in the text, $L_2$ the number of bigrams, and so on, and let $L = L_1 + L_2 + \ldots$ denote the total number of features in the text, where the sum thus extends to the maximum value of *n* in the feature set; typically $n=2$, as mentioned above. In order to classify a text, the following sum is computed

2 $$\begin{aligned} s = \alpha + \sum _{i=1}^{V}w_i f_i, \end{aligned}$$

where $\alpha$ is a bias term and $w_i$ are the feature weights that, as will be shown below, are initially in the range $[-1,1]$. It should be noted here that, even though *V* is typically quite large (millions), most feature values $f_i$ are zero, as only the features actually present in the text under consideration are effectively included in the summation. Once *s* has been computed, it is normalized by the number of features actually present in the text, i.e.,

3 $$\begin{aligned} {\overline{s}} = \frac{s}{1 + L_1 + L_2 + \ldots } \equiv \frac{s}{1 + L}. \end{aligned}$$

where the 1 accounts for the bias term $\alpha$. Finally, a length adjustment factor is added, to form the final *classification measure* $\sigma$

4 $$\begin{aligned} \sigma = {\overline{s}} + \beta (L_1). \end{aligned}$$

The classifier thus maintains a list of $\beta (L_1)$, one for each value of the text length. If the length of a classified text exceeds the maximum length $L^\mathrm{{max}}_1$ of any text in the training set, the value $\beta (L^\mathrm{{max}}_1)$ is used instead. The length adjustment gives a small, but positive, contribution to the classification performance, at least for the data sets considered here. Note that, if the length of the classified texts happens to be *irrelevant* for classification, the optimizer will set the corresponding parameters to near-zero values, thus effectively removing them from consideration.

Classification is then straightforward: If $\sigma \ge 0$, the text is classified as belonging to Class 1 (written text, in our case), otherwise it is classified as an instance of Class 0 (spoken utterances, here).

We remark here that the classification measure $\sigma$ itself has a dual purpose. It determines the class assignment as just described, but its magnitude, i.e., $\vert \sigma \vert$, can also act as a reliable measure of the classifier’s confidence in its own classification, as discussed below; see Sect. 5.3. Note also that the normalization in Eq. 3 is not really needed from a *classification* perspective (since the classification threshold is 0, and the length adjustment weights $\beta (L_1)$ could be re-scaled), but it is needed in order for $\vert \sigma \vert$ to be used as a confidence measure.

#### Generating and using the classifier

As can be seen above, our classifier is fully determined by the values of the bias term $\alpha$, the *V* weights $w_i$, and the length adjustment factors $\beta (L_1)$. When generating a classifier (as a precursor to optimization; see below), the bias term and the length adjustment terms are typically set to zero. The weights $w_i$ are initialized based on the number of instances of the corresponding feature $f_i$ in the two classes. Considering the training set, let $c_0(i)$ and $c_1(i)$ denote the number of instances of feature *i* in the texts (sentences, in our case) belonging to Class 0 and Class 1, respectively. The initial value of $w_i$ is then assigned as

5 $$\begin{aligned} w_i = \frac{c_1(i)-c_0(i)}{c_1(i)+c_0(i)}, \end{aligned}$$

resulting in values in the range $[-1,1]$, such that values close to 1 are indicative of a feature that predominantly occurs in Class 1, and values close to -1 signify a feature that chiefly appears in Class 0. Note that the values of $c_0(i)$ and $c_1(i)$ are available in the feature set by construction (see Sect. 4.1.3 above), so that a classifier can be generated very fast.

Once the classifier has been generated, its performance over any indexed data set (e.g. the training set or the validation set considered here) can be computed very quickly. For each feature $f_i$ that appears in a text under consideration, the corresponding index is available (as a result of the indexing step described above), so that the weight $w_i$ can simply be read off from the index. The length adjustment term can also easily be accessed, given information (which is stored as well) about the number of tokens in the text being classified. Thus, for the proposed classifier, the classification time complexity is *O*(*L*).

For a non-indexed text, such as when the classifier is actually used, after optimization, the text must first be indexed, something that involves a binary search over the features with time complexity $O(L\log {L})$ (for the *L* features, in total) followed by the actual classification with time complexity *O*(*L*) as above.

### Optimization

The optimization procedure is straightforward as well. During optimization, the performance over the validation set is computed first, using a suitable performance measure; here, the accuracy was used, i.e.,

6 $$\begin{aligned} a = \frac{\mathrm{{TP}} + \mathrm{{TN}}}{\mathrm{{TP}} + \mathrm{{FP}} + \mathrm{{TN}} + \mathrm{{FN}}}, \end{aligned}$$

where TP are the true positives, i.e., texts belonging to Class 1 that are actually assigned (by the classifier) to Class 1, TN are the true negatives, FP the false positives, and FN the false negatives.

Next, the performance is computed over the training set. For that set, the feature errors, defined as

7 $$\begin{aligned} e_i = \frac{1}{\gamma _i}\sum _{j=1}^{k}v_{ij}\left( {\hat{C}}_j-C_j\right) , \, i = 1,\ldots ,V, \end{aligned}$$

are computed, where $\gamma _i = c_0(i) + c_1(i)$ is the total number of instances of $f_i$ in the entire training set, $v_{ij}$ is the number of instances of feature $f_i$ in sentence *j*, whereas $C_j$ and ${\hat{C}}_j$ respectively are the true and inferred classes (represented as integers) for sentence *j*, i.e., either 0 or 1. After computing $e_i$ for all features, the weights are updated as

8 $$\begin{aligned} w_i \leftarrow w_i - \eta e_i, \end{aligned}$$

where $\eta$ is the learning rate. For the bias term, the error is instead simply defined as

9 $$\begin{aligned} e_{\alpha } = \frac{1}{k}\sum _{j=1}^{k}\left( {\hat{C}}_j-C_j\right) , \end{aligned}$$

and $\alpha$ is then updated as

10 $$\begin{aligned} \alpha \leftarrow \alpha - \eta e_{\alpha }, \end{aligned}$$

Finally, for the length adjustment terms, $\beta _i$, the error is computed as

11 $$\begin{aligned} e_{\beta , L_1} = \frac{1}{k_{L_1}}\sum _{j=1}^{k}\delta (L_1)\left( {\hat{C}}_j-C_j\right) , \end{aligned}$$

where $\delta$ is a delta function that takes the value one for the given value of $L_1$, and 0 for all other values, and $k_{L_1}$ is the number of texts with length $L_1$ tokens. Here, given that the length adjustment is added to the normalized sum ${\overline{s}}$ (see Eq. 4), a different (smaller) learning rate $\nu$ is used, such that

12 $$\begin{aligned} \beta (L_1) \leftarrow \beta (L_1) - \nu e_{\beta , L_1}. \end{aligned}$$

Once the new parameters (weights, biases, and length adjustments) are available, the first training epoch (iteration) is completed. Next, all the sentences are classified again, using the new parameters, after which new errors can be computed so that the parameters can be updated again, and so on, for any number of epochs. Optionally, the learning rate can be set to decay, by modifying it (after every training epoch) as

13 $$\begin{aligned} \eta \leftarrow \kappa \eta , \end{aligned}$$

where $\kappa$ is a positive constant, smaller than (or equal to) 1.

During optimization, holdout validation is used, in which both the training and validation sets are considered: The performance over the training set is used when updating the weights, whereas the validation performance is used for determining when to stop the training (but does not effect the parameters), so as to avoid overfitting. In other words, the best parameter set is taken as that which minimizes the error over the validation set.

Note that, while the learning rule bears some resemblance to the stochastic gradient descent learning rule that can be applied in linear (and logistic) regression, there are also differences, notably the fact that, in our method, the weight update is based on the local error, i.e., the contribution of each feature, in isolation. Moreover, the error uses the discrete class assignment (the inferred class vs. the ground truth class), rather than the classification measure, that is, the squared weighted feature sum, as would be the case in linear regression.

## Results

The method described above was implemented in C#.NET (i.e., *without* making use of ready-made functions for tokenization etc.). This was a deliberate choice in order to maintain flexibility over the various steps in the procedure.

The method was then applied to the training set presented in Sect. 3, considering unigrams and bigrams,^2^ i.e., with $n_\mathrm{{max}} = 2$. The tokenizer produced a set of 1,484,934 distinct tokens. The threshold *p* was set to 3, meaning that any feature with less than 3 instances (in the training set) was eliminated. The resulting feature set contained a total of 4,467,598 features, of which 481,452 unigram features and 3,986,146 bigram features. Next, the training, validation, and test sets were indexed, using the feature set defined for the training set. As mentioned above, while not strictly necessary, the indexing greatly increases the speed of the classifier. Then, a classifier was generated assigning initial weights as described in Sect. 4.2.1. Several runs where then carried out, using the optimization procedure described above, and with different values of the learning rates $\eta$ and $\nu$ and the decay rate $\kappa$. While the final results did not depend strongly on the values of the learning rate, the optimization time was found to be shortest with the values $\eta = 2$, $\kappa = 0.975$, and $\nu = 0.10$.

**Fig. 1:**
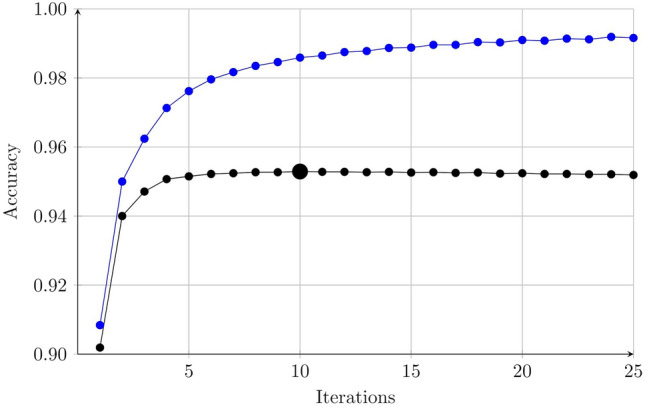
The performance of the classifier during the first 25 iterations of an optimization run with $\eta = 2$, $\kappa = 0.975$, and $\nu = 0.10$. The blue line shows the accuracy over the training set and the black line shows the accuracy over the validation set. The best classifier, with a validation accuracy of 0.9529 (marked with a bigger disc), was achieved in the $10^\mathrm{{th}}$ iteration

The performance over the validation and training set as functions of the number of training epochs, using the accuracy (Eq. 6) as the performance measure, is shown in Fig. 1. As can be seen from the figure, the best validation performance, an accuracy of 0.9529, was found after 10 epochs. The corresponding classifier was saved, and was then, finally, applied to the previously unused test set, resulting in an accuracy of 0.9561. The optimization run was carried out on a standard desktop computer with 32 GB RAM and an Intel Core i9 processor running at 3.1 GHz, using which a training epoch (i.e., computing the performance over the validation and training sets, and then updating the parameters) took around 22.4 s, meaning that the best validation score was found after less than 4 min. of running time. As further discussed in Sect. 6, the speed could quite easily be significantly improved though, but it was not deemed necessary to do so here. The main point is that the procedure can easily be carried out on standard hardware.

### Comparing classification methods

For the purposes of comparison, several other classification methods were tried as well. In all cases (including our method) the starting point was the same, namely the training and validation sets, described in Sect. 3, and the final evaluation was based on the performance over the test set described in the same section.

The methods considered were divided into three classes: (i) Classical methods, a category that here involves linear regression (with thresholding to generate a classifier), logistic regression, multinomial naïve Bayes, Bernoulli naïve Bayes, SVMs, a ridge classifier, and a decision tree; (ii) explicitly interpretable methods, a category that here contains only our method (see also Sect. 5.4); and (iii) black box methods, comprising a shallow neural network (NN) and a DNN (DistilBERT).

For the classical methods, the Python implementations in scikit-learn (v. 1.2.1) were used, along with its default tokenizer, defining a total of 972,896 unique tokens, i.e., a bit less than the number obtained with our tokenizer. In the case of multinomial and Bernoulli naïve Bayes classifiers, the default settings were used, i.e., with the additive smoothing parameter set to one, and prior probabilities adjusted according to the data. In the case of linear regression, an exact solution was generated via matrix inversion, rather than using stochastic gradient descent. For logistic regression, we used the SAGA solver. Similarly, for the SVM, the stochastic gradient descent method with hinge loss function was used (i.e., the SGDClassifier implementation with default parameters). In the case of the ridge classifier, the default settings were used, i.e., with the regularization coefficient set to one. A maximum depth of 35 was set for the decision tree classifier, which was generated using the CART algorithm.

For black box methods, we used the same tokenization as in the classical methods for the shallow NN, while for the DNN the tokenization follows the pre-trained BERT scheme, with a vocabulary size of 30,522 tokens in total (much fewer than in the other cases). The shallow NN is structured as an embedding bag with an embedding size of 64, plus a linear layer for classification. It has been implemented with PyTorch and trained with cross entropy loss, the SGD optimizer (running five epochs), and a batch size of 128. As for the structure, the DNN is the Hugging Face pretrained DistilBERT uncased,^3^ fine-tuned to our classification task with the training and validation sets (using batch size: 32, epochs: 5, learning rate: $2\times 10^{-5}$).

**Table 3:** Performance over the test set for several different methods

Classical methods						
	accuracy	precision	recall	F1	*r*	rank
Multinomial naïve Bayes	0.898	0.866	0.957	0.909	0.933	9
Bernoulli naïve Bayes	0.916	0.930	0.911	0.920	0.945	8
Linear regression	0.927	0.929	0.934	0.932	0.957	5
Logistic regression	0.922	0.913	0.944	0.928	0.953	7
Support vector machine	0.834	0.784	0.951	0.860	0.883	11
Ridge classifier	0.924	0.915	0.944	0.929	0.954	6
Decision tree	0.846	0.809	0.930	0.865	0.888	10

Explicitly interpretable methods						
	accuracy	precision	recall	F1	*r*	rank
**Our classifier**, $n_\mathrm{{max}}=1$	0.943	0.947	0.946	0.947	0.972	3
**Our classifier**, $n_\mathrm{{max}}=2$	0.953	0.951	0.961	0.956	0.982	2

Black box methods						
	accuracy	precision	recall	F1	*r*	rank
Shallow neural network	0.931	0.931	0.940	0.936	0.961	4
DNN DistilBERT	0.973	0.975	0.973	0.974	1.000	1

Note: For the classical methods, the unigram bag-of-words features were used, obtained with the scikit-learn tokenizer. For our method, two cases are shown, namely $n_\mathrm{{max}} = 1$ (using only unigrams as features) and $n_\mathrm{{max}} = 2$ (using unigrams and bigrams). In both cases, features were generated with our tokenizer, and were included only if they had at least 3 instances in total, in the training set. The column marked *r* measures the F1 score relative to the F1 score for DistilBERT, whereas the right-most column shows the ordinal rank of the different methods

The results over the test set are presented in Table 3. As can be seen from the table, focusing on the *F*1 score, the classical methods (for which unigram features were used) range from 0.860 to 0.932, with an average of 0.913, whereas the DNN-based classifier (DistilBERT) reaches an F1 score of 0.974, i.e., a performance boost of around 0.06, roughly in line with the findings from other studies, as discussed in Sect. 2 above. For our classifier, with $n_\mathrm{{max}} = 1$, i.e., using only unigrams as features, and applying the optimization method from Sect. 4.3, the result over the test set was an F1 score of 0.947, which is better than all the classical methods. For $n_\mathrm{{max}} = 2$, i.e., using unigrams and bigrams as features, the performance of our method, an F1 score of 0.956, is only less than 0.02 below the results from DistilBERT. This is an interesting result, in our view: With such a small performance difference, the choice of classifier comes down to the need (if any) for understanding exactly *how* the classifier arrived at its decision, something that our method easily offers (see Sect. 5.3 below), but which is *not* the case for black box methods, including DistilBERT.

While Table 3 offers a fair *relative* comparison of the different methods, in all cases using the same training, validation, and test sets, one should be careful not to draw too firm conclusions regarding the *absolute* classification accuracy: It is possible that artifacts in the data, or the fact that the data are derived from just a few sources (albeit large ones), may inflate the classification performance in a way that would not apply for new, unseen data.

### Running time comparison

Starting with the process of generating a classifier, one can divide it (for all methods) into two distinct steps: First, a preprocessing step is carried out, involving tokenization and indexing (or vectorization), which is rather similar for all methods, and can be carried out once and for all, for a given data set; see Sect. 4.1.3; Next, after preprocessing, the classifier is optimized. For the proposed method, with the data set considered here, each iteration of the optimizer lasted around 22 s so that the entire optimization run shown in Fig. 1 took around 9 min to complete, on a standard desktop computer. Note that, if needed, the optimization can be speeded up by around a factor three or so, by using the fact that the texts are classified independently of each other, meaning that the classifier could be modified to run over multiple processor cores. By way of comparison, the optimization time for the other classical methods were: A few seconds for naïve Bayes and SVM; a few minutes for logistic regression and the ridge classifier; about one hour for linear regression (executed in parallel over 24 cores); and about 5 h for the decision tree. For the black box methods, the optimization of the shallow NN took a few minutes, whereas fine-tuning DistilBERT took about 60 h. Note that the optimization time for the two methods based on neural networks is not directly comparable with the others since different hardware (GPU rather than CPU) was used in those cases.

Turning now to the use of the optimized classifier, running through the entire test set (with 1,948,631 sentences; see Sect. 3) took a few seconds with our method (and the classical methods), again using a standard desktop computer, whereas it took about 20 min with DistilBERT.

**Fig. 2:**
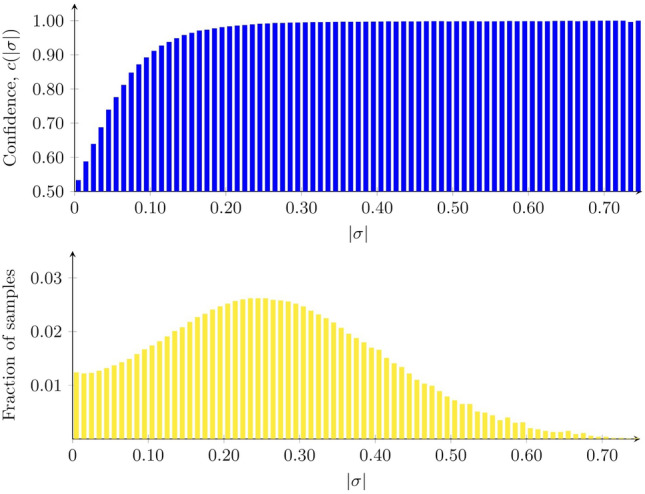
Top panel: The confidence histogram, computed over the validation set, using a bin width of 0.01. For each bin, the corresponding bar measures the fraction of correct classifications (whether in Class 0 or Class 1), i.e., the classification accuracy, for those texts whose value of $\vert \sigma \vert$ fell into the bin in question. Note that the histogram extends all the way up to $\vert \sigma \vert = 1$, but the fraction of samples with $\vert \sigma \vert > 0.75$ (for which the accuracy is equal to 1) is a tiny fraction of the total, and they are therefore not shown. Bottom panel: The fraction of samples in each bin. The bulk of the samples fall into bins for which the accuracy is very high; see also the main text

### Confidence measure

As it turns out, the absolute value of the classification measure, i.e., $\vert \sigma \vert$ (see Eq. 4), can be used as the basis for a measure of confidence, as follows: After optimization, the classification performance for the $k_\mathrm{{val}}$ texts in the *validation* set^4^ was used in order to form a confidence histogram, measuring the classification accuracy as a function of $\vert \sigma \vert$. The values of $\vert \sigma \vert$ were binned in intervals of width *d*. For each bin, the fraction of correct classifications were computed for those texts whose value (whether negative or positive) of $\vert \sigma \vert$ fell into the bin in question. This fraction is henceforth referred to as the *confidence*, denoted $c(\vert \sigma \vert )$, which is thus a local measure, pertaining to a narrow range of $\vert \sigma \vert$ values.

The resulting histogram is shown in the top panel of Fig. 2. As can be seen from the figure, the confidence rises very quickly with $\vert \sigma \vert$. The bottom panel of the figure shows the fraction of samples in each bin. From the data underlying these figures, one can compute the fraction of samples for which the accuracy exceeds a given threshold; it turns out that 74.4% of samples have $\vert \sigma \vert > 0.15$, where the accuracy is 0.97 or better. Similarly, 67.4% of samples fall in bins with $\vert \sigma \vert > 0.19$, for which the accuracy is 0.98 or better. Finally, 54.6% of samples have $\vert \sigma \vert > 0.24$, where the accuracy is 0.99 or better.

**Fig. 3:**
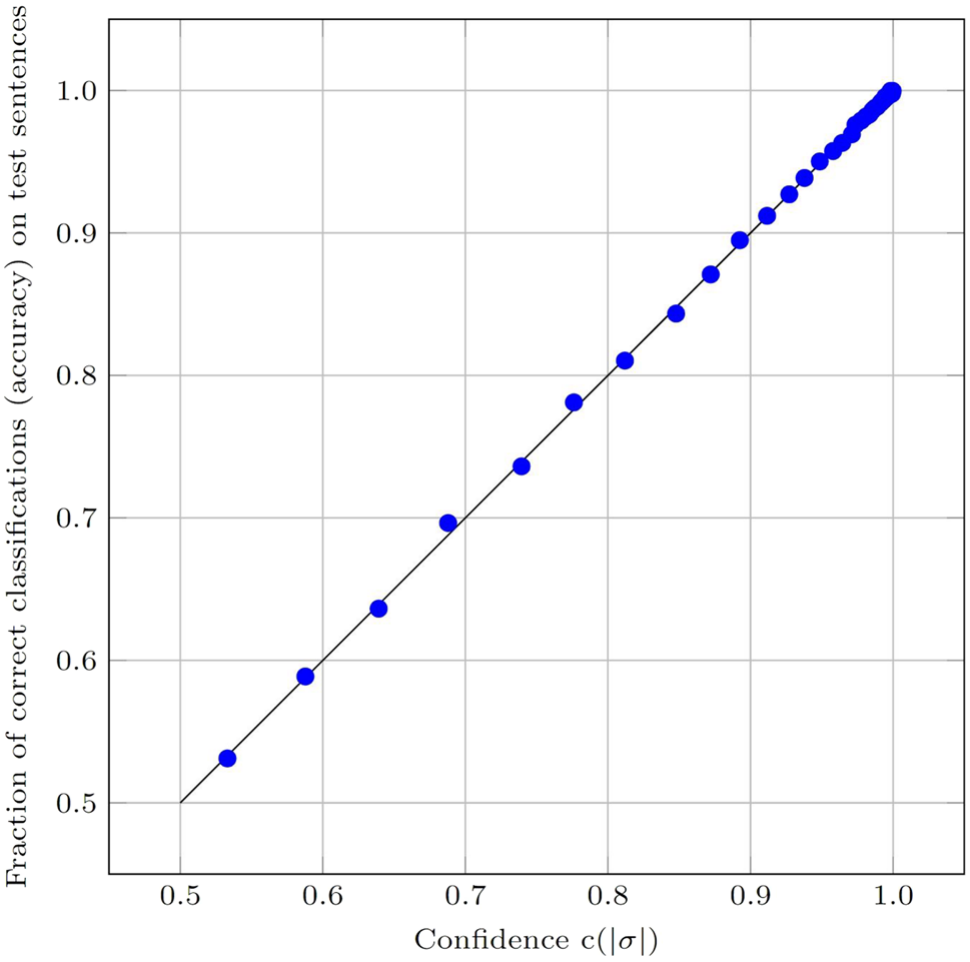
Fraction of correct classification on the test set for each confidence $c(\vert \sigma \vert )$ bin. Note that only the bins up to $\vert \sigma \vert = 0.5$ have been plotted. Beyond this point, the confidence values are near or at 1, for both histograms

Figure 3 illustrates that the confidence measure $c(\vert \sigma \vert )$ is reliable by showing its application to the test set. In this figure, every point represents one bin. The horizontal axis measures the expected confidence $c(\vert \sigma \vert )$ based on the validation histogram and the vertical axis shows the actual test set accuracy for the corresponding bin. As can be seen in the figure, $c(\vert \sigma \vert )$ matches almost perfectly the probability of classifying a test sentence correctly. As a specific example, consider the bin with values of $\vert \sigma \vert$ in [0.200, 0.210]. For the validation histogram, there were 49,041 sentences in this range, of which 48,232, or 98.35%, were correctly classified, so that the confidence was equal to 0.9835 for this bin. For the test set, 49,338 sentences fell in this bin, of which 48,511, or 98.32%, were correctly classified, giving an accuracy of 0.9832. The corresponding point is among the aggregation of points near the top-right in the figure.

**Fig. 4:**
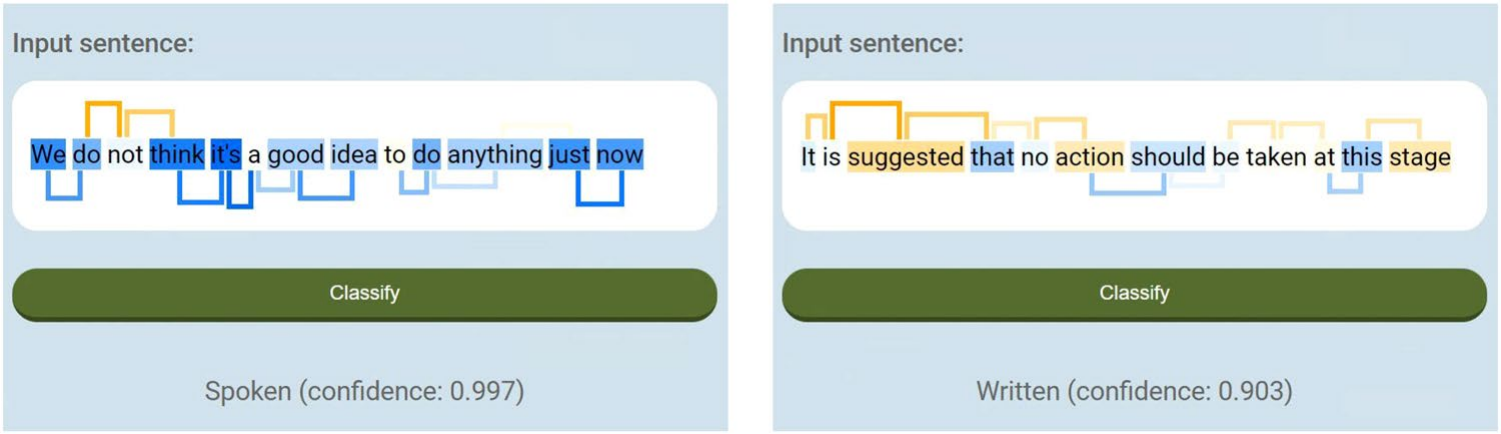
Two examples of the visualization of our classifier, obtained from the web-based tool at http://aaiserver.m2.chalmers.se/spoken_vs_written_tool. The left panel shows an informal (spoken) version of a sentence, and the right panel shows a formal (written) version of the same sentence. Both sentences are correctly classified, i.e., Class 0 for the first sentence and Class 1 for the second. Note that the visualizer can also (optionally) show the numerical values for the bias, the weights, and the length adjustment

### Visualization and interpretability

One of the main motivations behind this work has been to arrive at a classifier that exhibits high performance while, at the same time, offering a considerable degree of interpretability. To this end, we devised a visual representation of the classifier, two examples of which are shown in Fig. 4. As can be seen in the figure, the individual tokens (unigrams) are color-coded, such that those with negative weights (promoting classification into Class 0) are shown by highlighting the token in question in blue color, whereas positive weights (favoring Class 1) are associated with yellow-orange color. For the bigrams, the color coding is the same, but is indicated using a form of bracket, such that negative weights are shown below the text, whereas positive brackets are shown above. Moreover, the height of the bracket is proportional to the value of the weight, thus providing a second way of understanding the impact of a given bigram. The bias, feature weights, and length adjustment are also (optionally) shown in numerical form, making it possible to retrace the classifier’s entire computation. Finally, the class assignment is shown, along with the confidence measure, computed by linear interpolation over the histogram described in the previous section.

We have also generated a basic web-based tool for classifying sentences using the two classes described here. The tool, which makes use of the best classifier (F1 = 0.9561) and the validation histogram, is available at http://aaiserver.m2.chalmers.se/spoken_vs_written_tool. Note that, given the fact that written text is often more formal than spoken text, the classifier can be used, for example, as an aid in writing, to determine whether a given sentence adheres to a formal or an informal style.

We remark that the visualization described above is an exact representation of the computation carried out by the classifier. That is, for our classifier, there is no need to resort to approximate methods for visualization; see also Sect. 6.

### Fill-in-the-blank performance of DistilBERT

**Table 4:** Fill-in-the-blank results in terms of the probability of selecting the ground-truth word, for different levels of *spokenness*

Spokenness	$-$1.35	$-$1.05	$-$0.75	$-$0.45	$-$0.15	0.15	0.45	0.75	1.05	1.35
Average	0.136	0.284	0.296	0.232	0.209	0.186	0.229	0.234	0.289	0.142
Std. dev	0.248	0.362	0.356	0.323	0.307	0.291	0.314	0.301	0.341	0.250

Note: *spokenness* of the masked word vs. the probability of DistilBERT predicting the correct word. The *spokenness* values in the table represent the center of the bins. Results are shown in terms of average and standard deviation of the predicted probability of the correct word

Since BERT (and, by extension, DistilBERT) was trained mostly on written text, it may be that its ability for accurate classification comes down to an out-of-distribution detection, i.e., determining whether or not a given text is similar in its construction to the texts over which it was trained. In that case, its fill-in-the-blank performance might be worse for spoken text than for written text. In order to investigate this issue, as a first experiment we ran the pre-trained DistilBERT, without fine-tuning, over the test set, masking whole words randomly with a probability of 0.15. A resulting perplexity of 27.50 was obtained for Class 0 (spoken) and 20.96 for Class 1 (written) sentences, meaning that the classifier is slightly more accurate with written text, in this case.

However, as a more detailed, second experiment, we masked words according to their *spokenness* measure (see Eq. 1) and their frequency, to investigate whether DistilBERT performs better with words that are more common in written text. Specifically, we considered for masking only words with similar number of instances, in this case $10,000 \pm 2,000$, in the training set. Then, for each sentence (all of which contain at least 5 tokens, as mentioned above) in the test set, we masked a single word with *spokenness* in the range [$-$1.5, 1.5], so as to obtain a uniform distribution of masked words in that range, over the entire test set. The results of the model predictions, in terms of the probability of selecting the correct word, are listed in the Table 4. In summary, there is no clear correlation between the *spokenness* of masked words and the performance of the model.

## Discussion

The main motivation behind this work has been to determine whether a method derived from classical ones can achieve a performance at least approaching that of DNN-based methods. We have shown that, with careful choices of tokenization, feature selection, and the optimization method, it is possible to reduce the performance gap, at least for the data set considered here, to such a small value that the choice of classifier depends on the need for interpretability (if any) rather than performance considerations.

Comparing with the classical methods, our approach offers better performance, even when only unigram features are used, and even more so once bigram features are included as well. More importantly, the proposed method is associated with a confidence measure and an exact visualization tool, which are crucial components with regard to reliability and interpretability, respectively.

The contributions from the individual unigrams and bigrams towards a given class assignment are easy to determine from the visualizer (see Fig. 4 and the online tool), where also (optionally) the numerical weights for each feature can be shown. Since the features are combined linearly, it is also possible to assess how the overall classification is obtained from the individual components. This is in contrast to the transformer-based approaches, such as DistilBERT: Even though it is possible to visualize how the attention mechanism emphasizes certain tokens, the highly non-linear manner in which the values are then *used* renders the transformer opaque [22]. Furthermore, using another form of explanation method (e.g., LIME or SHAP [8, 9]) leads to an approximation of the transformer’s decision [23], while the explanation obtained by our approach is, by construction, precisely the computation performed by the classifier. Thus, we believe that our method, with the associated visualization tool and confidence measure, provides a deeper and more intuitive understanding of how the classifier arrives at a decision than explainability measures and other tools that are sometimes applied to DNNs [24].

**Table 5:** Results obtained (over the test set) for different values of the inclusion threshold *p*

$n_\mathrm{{max}} = 1$			$n_\mathrm{{max}} = 2$		
*p*	# features	F1	*p*	# features	F1
3	481,452	0.9466	3	4,467,598	0.9561
10	195,339	0.9452	10	1,456,495	0.9558
30	94,662	0.9436	30	584,505	0.9549
100	45,096	0.9413	100	219,376	0.9525

For a given value of *p*, features are included only if they occur at least *p* times in the *training* set. For each entry in the table, an optimization run was carried out, using the same learning rate parameters as in Sect. 5. The resulting classifier was then evaluated over the test set. The left part of the table shows the results obtained with $n_\mathrm{{max}} =1$, i.e., using only unigrams as features, whereas the right part of the table displays the results obtained for $n_\mathrm{{max}} = 2$, i.e., with unigrams and bigrams as features

As for the sentences that are misclassified by our method, we found that the sentence length is an important factor. Sentences with few tokens are more likely to be classified incorrectly than those with many tokens. In a short sentence, a single feature (unigram or bigram) may have a large impact on the assigned class. Thus, if a short sentence in one of the classes contains one or two features that are more frequent in the other class, the result may be an incorrect class assignment. In sentences incorrectly classified as belonging to Class 1 (written), such features include proper nouns, dates, and scientific terminology. In sentences misclassified as belonging to Class 0 (spoken), the crucial features are mainly pronouns, contractions of words, and interrogative tokens (question words as well as question marks). An additional interesting finding is that some of the sentences misclassified as Class 0 contain quotes or excerpts of dialogue. For those cases it is arguably the true class label that is wrong, rather than the label assigned by our classifier.

The proposed method can be applied to other text classification tasks, many of which might benefit from the confidence measure $c(\vert \sigma \vert )$. A possible application is in customer service, where an automated system may need to decide whether to handle a particular classification task (e.g., classifying incoming messages as a precursor to formulating an automated response) by itself, or send it onward to a human operator, a problem where the decision can be based on the confidence measure.

As mentioned in Sects. 4.1.1 and 5, our method uses many more features than is commonly generated by sub-word tokenizers, such as the tokenizer used in BERT. It is interesting to investigate how much of the performance boost of our method, relative to the classical benchmark methods (see Table 3), stems from the tokenization. To this end, several additional optimization runs were made, using different values of the inclusion cutoff *p*; see also Sect. 4.1.3. The results are presented in Table 5. As expected, the results drop as the number of features is reduced. However, the drop is quite small, and even for $n_\mathrm{{max}} = 1$ and with the smallest number of features (for $p=100$) our method still outperforms all of the classical methods, as well as the shallow neural network. We also reran our method, with $n_\mathrm{{max}} = 1$ and $p=3$, using the default scikit-learn tokenizer, which, as mentioned above, uses roughly two-thirds the number of tokens used by our tokenizer. The result was an F1 score of 0.9388, i.e., less than the F1 score obtained with our tokenizer.

The large token sets generated by our method may pose a severe problem for DNN-based methods, where the networks (already very large) would have to be scaled up (in size) by several orders of magnitude to accommodate such a token set. However, the method proposed here easily handles large token sets; as soon as the training and validation sets have been indexed (something that must be done only once), the computation time for classification is independent of the size of the token set, and the time required for weight adjustment (during optimization), already a small fraction of the total computation time, grows only linearly with the size of the token set.

## Conclusion

In this paper, we have introduced a method for text classification whose performance approaches that of a benchmark DNN-based method (DistilBERT) over a large data set involving spoken transcripts vs. written text, thus making the choice of a suitable method (e.g., ours or a DNN-based method) largely a question of whether interpretability is required. For the proposed method, a visual representation (available as an online tool) has been defined, making it possible even for a non-expert to understand why a given text is classified as belonging to a particular class. Moreover, in our method, the scalar value that underlies the classification also acts as a measure of confidence, a measure whose validity has been confirmed over the test set considered here. Finally, as a secondary point, we also investigated the performance of DistilBERT on fill-in-the-blank tasks, finding that the DNN performs similarly on written and spoken text, even though written text represents the majority of its training data.

## Data Availability

The data used when preparing the manuscript can be downloaded at https://doi.org/10.5281/zenodo.7694423.

## Footnotes

1 The data set can be downloaded at https://doi.org/10.5281/zenodo.7694423.

2 The case $n_\mathrm{{max}} = 3$ was considered as well, but the results obtained were slightly worse than those reported below.

3 https://huggingface.co/distilbert-base-uncased.

4 The validation set was used here, to avoid inflating the confidence score, keeping in mind that the accuracy over the training set is quite a bit higher than that obtained for the validation set; see also Fig. 1. For unseen data, the validation histogram provides a much better estimate of the actual confidence, as evidenced by the fact that the histogram for the *test* set (not shown here) is very similar to the validation histogram, as expected, given the very similar classification performance over those two sets; see also Fig. 3.

## Code availability

An online tool that implements our best text classifier can be found at http://aaiserver.m2.chalmers.se/spoken_vs_written_tool. The program code for generating and optimizing a classifier is available from the authors upon request.
